# Press Hardening of High-Carbon Low-Density Steels

**DOI:** 10.3390/ma18225163

**Published:** 2025-11-13

**Authors:** Filip Votava, Ludmila Kučerová, Štěpán Jeníček, Radek Leták, Jiří Hájek, Zbyšek Nový

**Affiliations:** 1 Regional Technological Institute, University of West Bohemia, Univerzitní 8, 301 00 Pilsen, Czech Republic; skal@fst.zcu.cz (L.K.);; 2 COMTES FHT a. s., Prumyslova 995, 334 41 Dobrany, Czech Republic; jiri.hajek@comtesfht.cz (J.H.); zbysek.novy@comtesfht.cz (Z.N.)

**Keywords:** low-density steels, press hardening, hot stamping, tailored properties, heat treatment, retained austenite, duplex steel, light-weight components

## Abstract

In this study, sheets of experimental high-carbon low-density steels (LDSs) with a thickness of 1.7 mm were processed in a combined tool designed for press-hardening. Press hardening, also known as hot stamping or hot press forming, is a manufacturing process used to create car body parts with exceptional mechanical properties and safety standards. These components often require tailored properties, meaning different mechanical characteristics in various parts of the component. LDSs have a lower specific density than conventional steels, so their use would be particularly suitable in automotive applications. Combined tools achieve distinct mechanical properties within a single part through thermomechanical processing. Simultaneous forming and heat treatment create tailored zones of high strength and ductility within the sheet metal. The hardened zone provides crashworthiness, while the more ductile zone absorbs kinetic energy and converts it into deformation energy. Hot stamping enables forming complex geometries from high-strength sheets with limited cold formability, a capability that can also be exploited for the aluminium-alloyed LDS under investigation in this work. Three different high-carbon LDSs with differences in chemical composition were subjected to this experiment, and the hardness, microstructure, and mechanical properties of the two areas of each sheet were evaluated. The aim is to determine their suitability for processing by press hardening and to try to achieve tailored properties (i.e., differences in ductility and strength across one part) as in a typical representative of 22MnB5 boron steel, where a strength limit of 1500 MPa at 5% ductility is achieved in the cooled part and 600 MPa at 15% in the heated part. Tailored properties were also achieved in the investigated LDS, but with only relatively small differences between the two tool areas. The omega profiles were produced by press hardening without visible defects, and it was possible to process the steels without any difficulties.

## 1. Introduction

Low-density Fe-Mn-Al-C-based steels are an emerging class of construction materials for application, particularly in the automotive, chemical, and aircraft industries. Their attractive combination of properties, including high energy absorption capacity, exceptional strength and toughness at both room and low temperatures, as well as excellent fatigue performance and elevated oxidation resistance, has sparked significant interest over the past decade. These steel grades hold promise for applications in lightweight, crash-resistant automotive body structures and structural components [1]. In general, LDS can be classified into four groups, according to their hot-rolled microstructures with a typical composition range:

- ferritic (Al ~ 5–9%, Mn < 5%, C < 0.05%),
- ferrite-based duplex (Al ~ 3–7%, Mn ~ 2–12%, C ~ 0.05–0.5%),
- austenite-based duplex (Al ~ 5–10%, Mn ~ 5–30%, C ~ 0.4–0.7%),
- austenitic (Al ~ 5–12%, Mn ~ 12–30%, C ~ 0.6–2.0%) [2].

**Figure 1:**
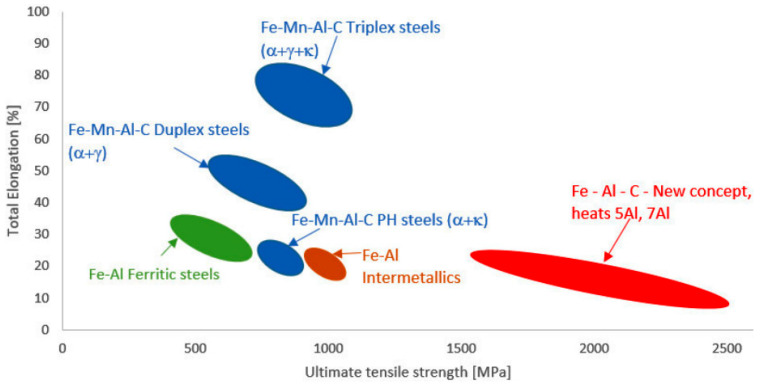
Banana diagram (Strength-elongation) of different groups of LDS [6].

This article uses an experimental press-hardening process to try to achieve tailored properties in the LDS group with a new alloying concept. Figure 1 shows the distribution of mechanical properties for the processed steels, represented by the red-shaded region corresponding to the highest ultimate tensile strength (UTS) values. These results are compared to other steel groups exhibiting varying chemical compositions. The new concept for alloying processed steels is described in detail in [3], and these steels are further investigated in this paper. Building on the findings reported in [3,4], these materials can be more accurately classified as Fe–Al–C-based steels compared with the typical chemical compositions discussed above. Their chemical composition has been slightly modified to enable effective heat treatment. The newly proposed steels are designed to fill the gap in alloys with medium to high carbon content, low manganese content, and aluminium levels between 5–12%. These variants are intended to serve as martensitic, hardenable, ultra-high-strength steels with reduced density. Owing to their lower alloying content, they are expected to be metallurgically simple to produce and cost-effective. Their high strength further offers the potential for material savings and additional weight reduction. Adding aluminium to steels leads to the stabilisation of the ferrite and forming undesirable intermetallic phases, which increase with higher aluminium content in the steel, significantly increasing when Al content exceeds 6.5 wt. %. The solubility of aluminium in the solid solution is limited. A detailed description of the occurrence of inter-metallic phases in the Fe-Al system is provided in [5]. To enable the practical use of Fe-Al-based alloys, it is necessary to add a suitable amount of austenite-forming alloying elements, such as Mn, C, and Cr, with which we can achieve a fully austenitic structure at higher temperatures, which is a prerequisite for further heat treatment [6].

Recent research has broadened the understanding of Fe–Mn–Al–C-based low-density steels (LDS), reinforcing their potential for lightweight structural applications. Zambrano et al. [7] provided a detailed overview of phase development and mechanical behaviour, emphasizing how duplex microstructures contribute to a favourable combination of strength and ductility. Ding et al. [8] demonstrated that austenitic LDS variants can achieve high energy absorption and tensile strength through mechanisms such as twinning-induced plasticity (TWIP). Kang et al. [9] showed that aging treatments at moderate temperatures can significantly enhance mechanical properties, with ultimate tensile strength approaching 900 MPa while maintaining excellent ductility. Sohrabizadeh et al. [10] investigated the tribological performance of aged LDS, revealing improved hardness and wear resistance—key factors for automotive components exposed to friction and dynamic loads. García-Domínguez et al. [11] explored the effects of microalloying with vanadium, finding that vanadium carbides contribute to grain refinement and increased hardness, supporting the development of steels with tailored microstructures. Kim et al. [12] offered a foundational review of Fe–Al–Mn–C alloys, discussing how aluminium content influences phase stability and density reduction, which are critical for designing cost-effective, high-performance steels suitable for automotive and aerospace applications.

Using LDS, car manufacturers could reduce body weight and fuel consumption even more while possibly improving crash safety, making the material ideal for safety components in car bodies [13]. The automotive industry is striving to maximise sustainability across all sectors. A notable example of this effort is the substantial expansion of electric vehicle production to reduce CO_2_ emissions. Additionally, minimising the weight of vehicle components can directly contribute to decreased fuel consumption, lowering the volume of exhaust gases generated by internal combustion and diesel engines. In the context of electric vehicles, weight reduction also translates to reduced electricity consumption [14]. A weight reduction of 100 kg is estimated to lower CO_2_ emissions by approximately 8.5 g per km [15]. Studies show that reducing vehicle weight by 10% can improve fuel efficiency by approximately 6% and increase the driving range of electric vehicles by up to 14% [16]. Furthermore, for conventional vehicles, every 100 kg of weight reduction saves approximately 0.19–0.32 L of fuel per 100 km [17].

The process of press-hardening is employed to fabricate high-strength components (e.g., as in a typical boron steel 22MnB5 reaching above 1500 MPa). It is characterised by its ability to shape sheet stock composed of hardenable materials using comparatively low forming forces, resulting in reduced springback tendencies [18]. Press-hardened components featuring tailored properties can avoid particular problems, such as two different materials being welded together, the presence of a weld, heat-affected zones (HAZ), and during crash test conditions when using a material with local soft areas. Furthermore, using tailored blanks in press-hardening facilitates the production of intricate geometries characterised by varying thicknesses and strength levels [19,20].

However, despite their advantages, the processability of LDS by press-hardening—particularly with tailored properties—has not been systematically investigated. This knowledge gap is what has motivated the present study. This study aims to investigate the production of small and large omega profiles through press-hardening of LDS, with the potential inclusion of subsequent heat treatment. These experiments aim to contribute novel insights into the press-hardening process, focusing on optimising process parameters and exploring the applicability and performance of these steels. Furthermore, the relationships between the processing parameters, the measured mechanical properties, and the observed microstructure of materials under investigation were examined.

To the best of our knowledge, no systematic study has yet been published on the applicability of press-hardening to Fe–Al–C steels with high carbon content and reduced manganese levels. Previous research has primarily focused on Fe–Mn–Al–C alloys or conventional boron steels (e.g., 22MnB5), leaving a gap in understanding how aluminum-alloyed LDS behave under industrially relevant hot stamping conditions. This work addresses that gap by evaluating three compositions (7SiAl, 7Al, and 5Al) in a combined tool designed for tailored properties, providing comparative data on microstructure evolution, mechanical performance, and processing feasibility. These findings establish a foundation for further optimisation and potential implementation of lightweight, high-strength Fe–Al–C steels in automotive applications.

## 2. Experimental Programme

### 2.1. Materials

Three high-carbon LDS (Low-Density Steels) (COMTES FHT a. s., Dobřany, Czech Republic) were used for the experiment. The experiment consisted of two parts. In the first part, the sheets were processed in a small omega tool with a larger number of samples to determine the appropriate heat treatment regime for the second part of the experiment, in which the sheets were processed in a combined tool (i.e., a large omega tool). All sheets had a thickness of 1.7 or 1.8 mm, and the chemical composition of individual melts is shown in Table 1.

Semi-finished products and samples of LDSs were sourced from ingots weighing approximately 50 kg. The melting and casting process for LDS is distinguished by the extensive use of aluminium as an alloying element and the deoxidising agent, ensuring that all the oxygen in the steel is fully bound. Consequently, the use of additional deoxidising agents is unnecessary. The significant deoxidation achieved with aluminium generates a substantial volume of slag, which must be removed during the melting process. Additionally, the process involves argon bubbling, which facilitates slag removal and aids in the capture of hydrogen and nitrogen. Argon also serves as a protective atmosphere throughout the procedure. These ingots underwent initial cutting at the top and bottom before being subjected to a homogenisation process at 1130 °C for eight hours, simultaneously heated in a furnace with two holds at 550 °C and 870 °C. Subsequently, they were heated to 1180 °C and underwent forging, and then hot rolling at 1180 °C at 20% reduction, which involved multiple passes to achieve a final thickness of 14 mm. A detailed description of the processing of semi-finished products is presented in [3,6].

Following descaling, the sheets were cold rolled with a 10% reduction to achieve a 1.8 ± 0.05 mm thickness. The rolled sheets were then subjected to annealing at 900 °C for 2 h, then cooled in a furnace. Lastly, the resulting sheet metal blanks were cut into the desired dimensions by waterjet (100 mm × 80 mm for the small tool, and approx. 160 mm × 160 mm for the combined tool) and then ground to a final thickness of 1.7 mm for the combined tool and 1.8 mm for the small tool. As can be seen from the description of the manufacturing process, the sheets are without further coating. The initial microstructures of the steels before processing consisted mainly of lamellar pearlite and ferrite, which contained a tiny proportion of carbide phases. A description of the structures was detailed in [3], where a new alloying concept for LDS was introduced.

The specific weight of these steels is: 7.18 g/cm^3^ for 5Al, and for steels with higher Al content (both 7Al steels), approximately 7.08 g/cm^3^ [6]. Compared to the average reported density of 22MnB5 (i.e., the best-known representative of press-hardening steel), which is approximately 7.8 g/cm^3^, a potential weight reduction of approximately 9% can be achieved according to our measurements. Other publications refer to similar weight savings when using steels with very similar chemical compositions. In [21], a saving of 8% is reported; in [22], a density reduction of 10% is claimed.

### 2.2. Small Omega Tool

**Figure 2:**
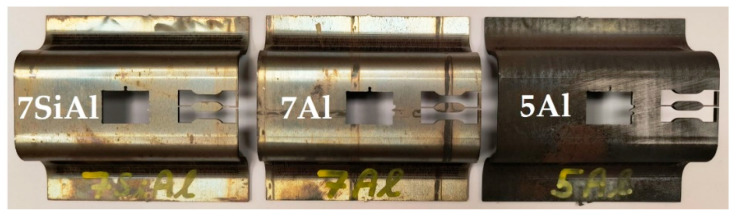
LDS small omega-shaped sheets.

Sheets with a size of 100 mm × 80 mm and a thickness of 1.8 mm were processed in the small omega tool. It is a specially designed tool to process sheet metal at elevated temperatures. The tool can be heated up to 450 °C. This heating is provided by heating cartridges at the top and bottom of the tool. The tool was mounted in a CKW 6000 hydraulic forging press (ŽĎAS, a.s., Žďár nad Sázavou, Czechoslovak Socialist Republic). After processing, an omega-shaped sheet profile is achieved, as seen in Figure 2. The tool is described in more detail in [23], where TRIP steels were processed.

Four different processing regimes were designed (described below) and were implemented for all three LDS, as shown in Table 2.

**Figure 3:**
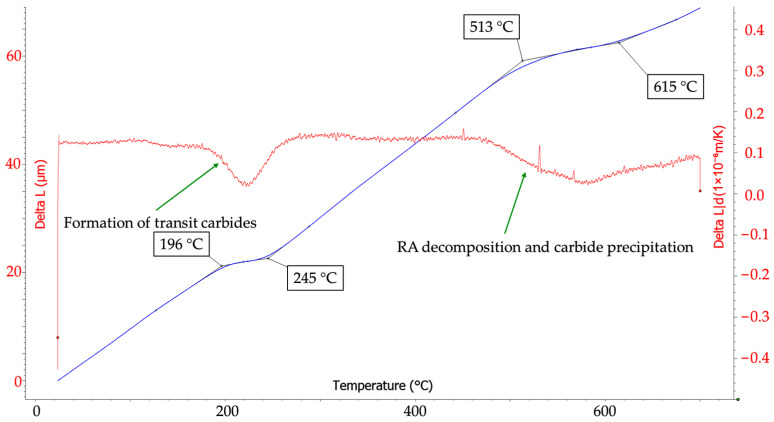
Tempering dilatometric curve for 5Al—heating up at 3 °C/min.

The experiments were preceded by calculations using JMatPro software, version 12.1, (Sente Software Ltd., Guildford, UK) to determine the required thermo-physical data [24]. Based on these calculations, a combined austenitization temperature of 950 °C was determined. The determination of tempering temperatures was based on the findings reported in [6] and on dilatometric measurements. Figure 3 illustrates the dilatometry curve for 5Al steel, indicating the onset of transition carbide formation until a temperature of approximately 250 °C is reached, and the decomposition of retained austenite is accompanied by carbide precipitation mostly under 600 °C.

Samples for metallography were cut from the omega-shaped sheets using a water jet to avoid creating a thermally affected area, and samples for the mini tensile test were taken by electro-erosive machining and ground to a thickness of 1.2 mm, as shown in Figure 2. The samples were analysed using an Olympus light microscope (Olympus Corporation, Tokyo, Japan), and Vickers hardness tests were conducted using a 10 kg load (LECO Instrumente Plzeň, spol. s r. o., Pilsen, Czech Republic). Tensile testing was carried out according to ČSN EN ISO 6892-1 method A [25], using an MTS E43.104 machine (MTS Systems Corporation, Eden Prairie, MN, USA) on mini tensile specimens with an active length of 5 mm and a cross-section of 2 mm × 1.2 mm.

The most suitable mode for sheet metal processing in the combined tool was selected based on the measured hardness values and the tensile tests conducted on samples processed in the small tool.

- The 7SiAl in the four modes achieved hardnesses ranging from 343 to 376 HV10, Rm ranging from 1086 to 1210 MPa, Rp0.2 ranging from 726 to 919 MPa, and ductility ranging from 3.9 to 7.5%.
- For 7Al, the measured values ranged from 326 to 377 HV10, Rm 1031 to 1245 MPa, Rp0.2 539 to 898 MPa, and ductility 4.1 to 8.3%.
- In the case of 5Al, 470 to 669 HV10, Rm 857 to 2023 MPa, Rp0.2 796 to 1545 MPa, and ductility from 0.7 to 6.6% were obtained.

**Figure 4:**
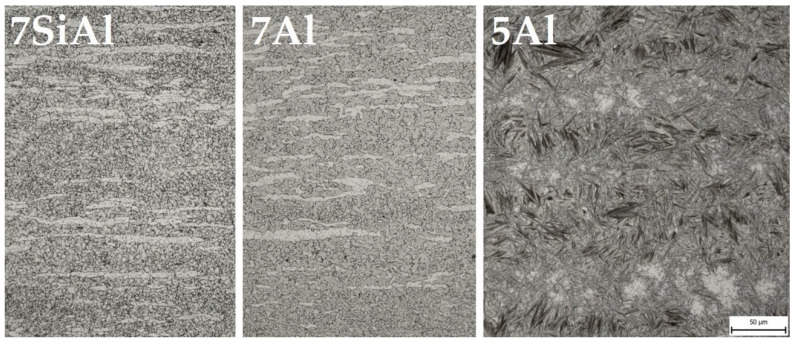
Microstructures after press-hardening in the small tool and 300 °C for 2 h in a furnace under an optical microscope. Magnification 500×. The 7SiAl and 7Al steel microstructure reveals fine austenitic equiaxial grains and numerous ferritic islands elongated in the direction of preliminary forming. The carbide phase is visible in both austenite and ferrite areas. 5Al steel reveals coarse needle martensite with a high amount of retained austenite.

In all three cases, the highest ductility values were obtained for the first mode (see Table 2), in which the sheets were subjected to a subsequent heat treatment at 300 °C for 2 h. Even in the case of 5Al, both the highest ductility and the Rm and Rp0.2 limits were achieved by this mode, which could be related to the brittle behaviour of the test specimens of the other modes during tensile testing. Therefore, the mode of subsequent heat treatment at 300 °C for 2 h was selected. Figure 4 shows the microstructures of the selected mode for all three materials. 7SiAl and 7Al steels consist of a mixture of ferrite and austenite; 5Al is a mixture of martensite and austenite.

Table 3 shows the measured values of the selected regime of the three LDS.

### 2.3. Combined Tool

**Figure 5:**
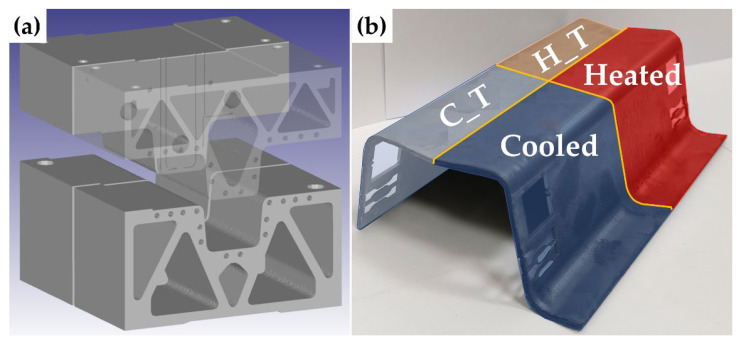
(**a**) Combined tool model; (**b**) Final shape of the omega profile from the combined tool. Steel 7Al (cut and sampled). The cooled part (C) is in the foreground and the heated part (H) in the background. The left part was subjected to tempering (T), and the right part was left untreated after press-hardening.

A special combined tool was used for the second part of the experimental programme shown in Figure 5a. This tool is specifically intended for tailored press-hardening, where one part of the tool is actively cooled while the other is heated. The final output obtained from this process is a sheet that adopts the form of an omega profile, shown in Figure 5b. The initial dimensions of the sheets were approximately 160 mm × 160 mm, with a thickness of 1.7 mm. For a comprehensive understanding of the tool’s construction and specifications, detailed information can be found in [26,27]. The tool was also mounted in the CKW 6000 hydraulic forging press, the same as the small one.

The processing parameters are shown in Table 4. The temperature of the heated part of the tool was set above the martensite start temperature (Ms) to suppress martensitic transformation during the dwell time in the die. However, after removal from the tool and subsequent cooling in air, martensite formation still occurred in the 5Al steel due to its particularly low Ms (~29 °C). Temperatures were calculated using JMatPro software (version 12.1). The calculated Ms values were: 229 °C for 7SiAl, 265 °C for 7Al, and 29 °C for 5Al. Then a slightly higher temperature (approx. 20 °C) of the heated part was chosen, shown in Table 4.

Due to the small number of sheets for the experiment, after processing, the sheet was cut in half longitudinally using a water jet, and the half (containing both parts, i.e., cooled and heated) was subjected to subsequent tempering (300 °C, 2 h) based on the findings obtained on the sheets processed in the small omega tool. For better clarity, the individual parts of the sheet were marked on the actual omega profile, as illustrated in Figure 5b.

### 2.4. Different Heat Treatment Parameters

A laboratory electric chamber resistance furnace, LAC LH 30/13 (LAC, s.r.o., Židlochovice, Czech Republic), was used to heat the sheets. Austenitization was carried out at 950 °C for 10 min to ensure full austenitization and temperature uniformity for all experiments. The sheets were then transported to the combined tool, which took approximately 5 s. The heated part of the tool was set up according to Table 4. The temperature of the cooled part was approximately 20 °C. Closing of the tool was done with a press speed of 100 mm/s, and it took 2 s. Sheet dwell times of 10 and 30 s were selected based on JMatPro simulations, ensuring compatibility with industrial processing limits. After processing, the sheets were removed from the tool and cooled freely in air.

### 2.5. Equipment Used for Evaluation

Metallography and mini-tensile test sampling were done from two areas of both halves of the sheet (i.e., press-hardened; press-hardened + tempered): cooled (C) and heated (H). The specimens were precisely sectioned using a water jet (for metallography) and electro-erosive machining (for mini-tensile tests) to mitigate the formation of a thermally affected area.

The samples for metallography were prepared by grinding, polishing, and etching in Nital (3%). The 5Al samples after press-hardening had to be etched in Vilella-Bain, as the images were not good after etching in Nital. The samples were observed on an Olympus light microscope (Tokyo, Japan). Scanning electron microscopy (SEM), Tescan VEGA 3 (TESCAN, s.r.o., Brno, Czech Republic), and Zeiss Crossbeam 340-47-44 (Carl Zeiss AG, Oberkochen, Germany) were used for detailed microstructure analysis. X-ray diffraction analysis was performed to determine the presence of phases and their quantification. The measurement was performed on a Bruker Discover D8 diffractometer (Bruker AXS, Karlsruhe, Germany).

Also, Vickers hardness measurements (LECO Instrumente Plzeň, spol. s r. o., Pilsen, Czech Republic) were performed with a load of 10 kg.

The tensile testing was performed according to ČSN EN ISO 6892-1 method A [25] on an MTS E43.104 machine (Eden Prairie, MN, USA) on mini tensile specimens with an active length of 5 mm and a cross-section of 2 mm × 1.2 mm. The final values are always the average of two tensile tests.

## 3. Results

### 3.1. Microstructure Analysis

**Figure 6:**
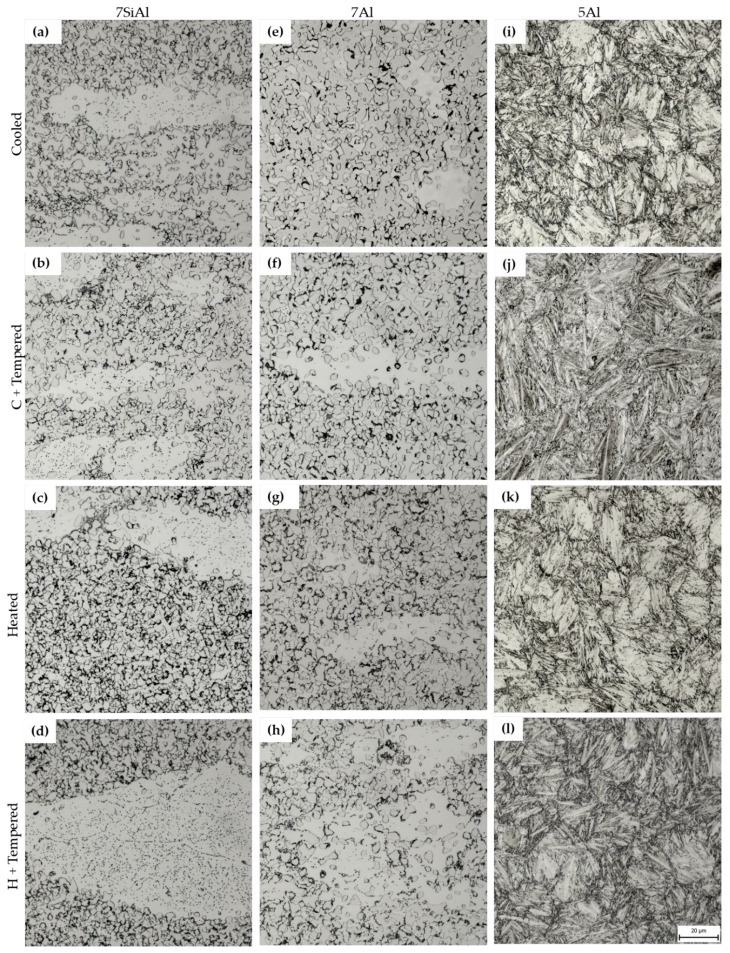
Light microscopy of LDS with a dwell time in the tool of 10 s. Magnification 1000×. 7SiAl (**a**–**d**); 7Al (**e**–**h**); 5Al (**i**–**l**).

**Figure 7:**
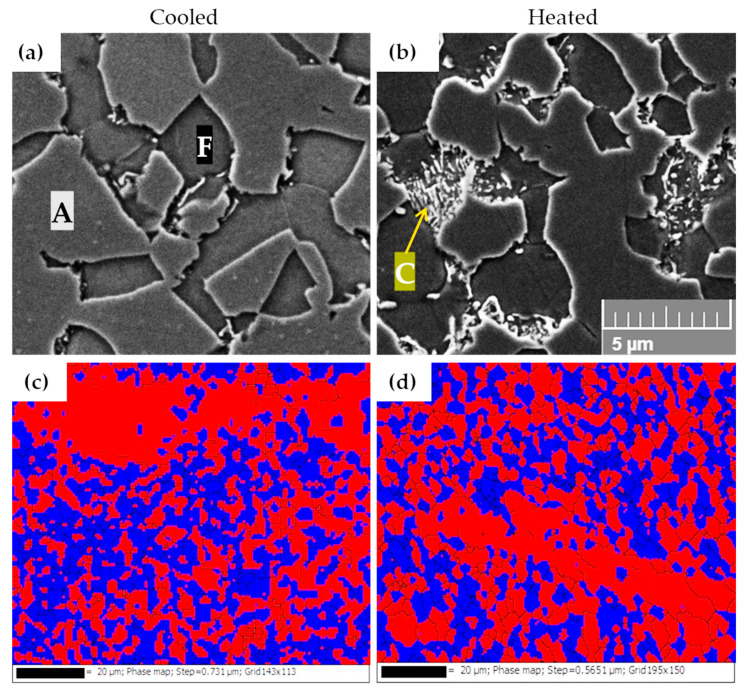
SEM (mag. 5000×) of 7Al steel (samples: dwell time 10 s without tempering) (**a**) cooled—ferrite (F) + austenite (A); (**b**) heated—ferrite + austenite + ferritic–carbide eutectoid/carbides (C); including EBSD (**c**) cooled; (**d**) heated. Red = BCC; blue = FCC.

The microstructure of the 7Al and 7SiAl steels after hot stamping with a 10 s dwell in the tool consisted of a mixture of ferrite, austenite, and carbides (Figure 6a–h). Light microscopy revealed a duplex matrix, in which elongated islands with reduced austenite content appeared as remnants of ferritic bands. EBSD and X-ray diffraction confirmed the high proportion of austenite, reaching up to 55% of the total volume (Figure 7c,d). While austenite takes the form of fine equiaxed grains, ferritic formations are coarse and elongated.

Higher magnification using SEM showed that austenite decomposed to a limited extent during press-hardening either into a ferritic–carbide eutectoid or discrete carbides (Figure 7a,b). The extent of this transformation depends on the cooling conditions. Material processed in the heated part of the tool exhibited a slightly higher amount of pearlite-like eutectoid, reflecting slower cooling rates that allow more time for austenite decomposition. By contrast, the cooled part contained fewer eutectoid blocks but a higher amount of fine, individual carbides. These distinctions were evident both in light microscopy (Figure 6a–h) and in detailed SEM observations (Figure 7a,b). Pearlitic blocks exhibited lamellar as well as granular morphologies. In the heated part of the 7Al steel, slightly larger globular carbides precipitated along ferrite grain boundaries and, to a lesser extent, within ferrite grains.

Dwell times of 10 s and 30 s played only a minor role in the final microstructure. For both conditions, using an initial sheet of 160 mm × 160 mm, the microstructural outcomes were comparable, indicating that the shorter dwell time is sufficient for processing. Minor differences were observed in the extent of carbide precipitation, with carbides appearing either as lamellar or granular eutectoid structures or as discrete particles; however, these variations did not substantially alter the overall microstructural features.

Comparing the high-Al steels, 7Al steel contained fewer purely ferritic elongated regions than 7SiAl steel, with fragmented austenitic islands more densely distributed. In the cooled section of 7Al steel, carbides were largely confined within pearlitic nodules, and no free carbides were detected outside these regions. Samples after tempering showed almost no difference from those with no tempering, see Figure 6a–h.

**Figure 8:**
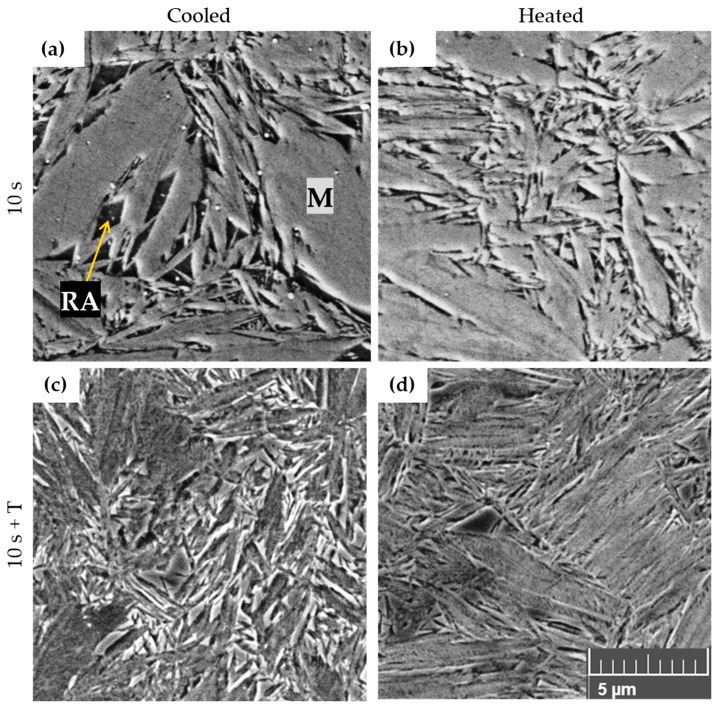
SEM of etched 5Al steel; dwell time 10 s. (T = tempering). Magnification 5000×. Etchant: (**a**,**b**)—Vilella-Bain; (**c**,**d**)—Nital. Martensitic matrix (M) and retained austenite (RA).

The lower aluminium and higher carbon content in the 5Al steel resulted in a distinctly different microstructure compared to the high-Al grades, characterised by a martensitic matrix with carbide particles, and retained austenite in all samples examined (Figure 6i–l). Fine globular particles were observed randomly dispersed within the martensitic laths in the as-quenched state. In samples processed in the cooled part of the tool, relatively coarse polygonal prior austenite grains (PAG) with diameters ranging from 5 to 20 μm were clearly visible, with larger grains occasionally detected. The PAG boundaries were sharply etched and represented weak regions prone to crack initiation and propagation. Some of these boundaries revealed signs of local failure, which may be associated either with stress accumulation during quenching or with the presence of intermediate phases along the grain boundaries. Larger cracks were frequently observed along PAG boundaries, while finer cracks appeared within the martensitic laths themselves (Figure 8a). By contrast, samples processed in the heated section of the tool did not show crack formation and exhibited a slightly finer martensitic microstructure (Figure 8b).

Within the PAG interiors, the martensitic laths contained a significant fraction of retained austenite and internal features that may correspond to deformation twins formed during rapid cooling. Rare, coarse spheroidised carbides up to about 3 μm in diameter were also present, indicating incomplete dissolution during austenitization.

**Figure 9:**
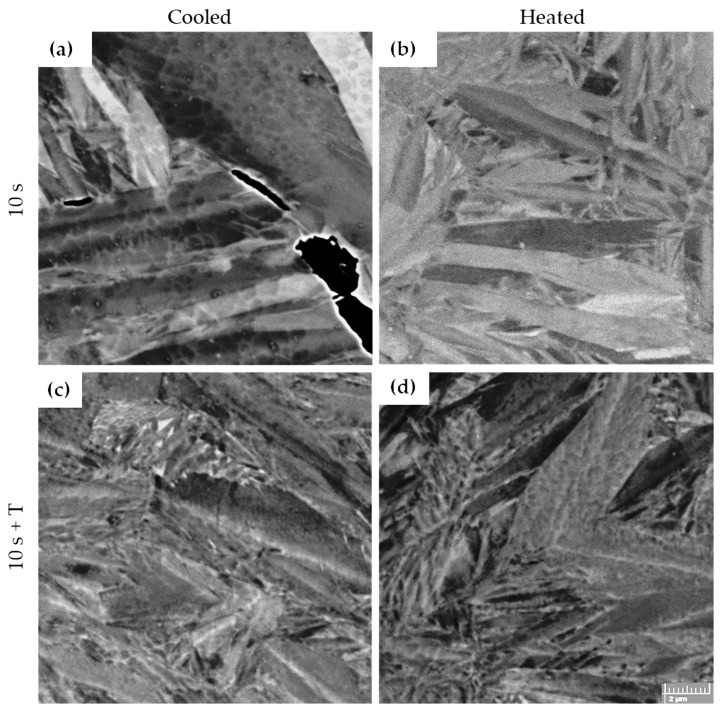
BSE of unetched 5Al steel; dwell time 10 s. (T = tempering). Magnification 10,000×. Martensitic microstructure (**a**,**b**), in (**a**) crack along the PAG boundary. Finer martensite laths in (**c**,**d**).

Tempering at 300 °C causes the formation of a substructure in the interior of the martensitic laths (Figure 8c,d). Residual austenite began to decompose, becoming more clearly delineated against the martensitic matrix. At the same time, fine lamellar features appeared within the martensitic laths, which are likely a combination of deformation twins and transition carbides (Figure 9a–d). Despite these changes, the overall microstructural characteristics of samples tempered after hot stamping in the heated and cooled parts of the tool remained very similar. The most evident tempering effects were the initial decomposition of retained austenite and the precipitation of fine transition carbides within the laths.

### 3.2. Mechanical Properties

Hardness measurements and mini-tensile tests were carried out as described above. These tests were performed for all processing conditions, with samples taken from the cooled (C) and heated (H) parts of the sheets.

**Figure 10:**
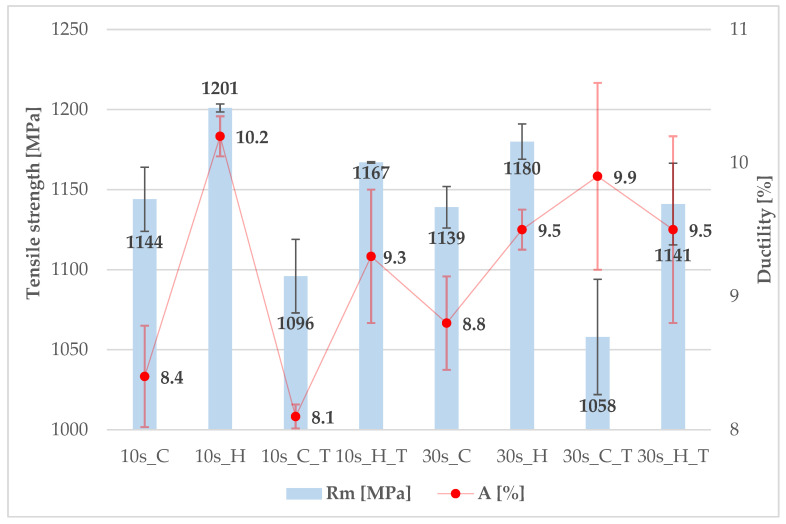
Mechanical properties of 7SiAl steel (cooled (C) and heated (H) part with or without tempering (T)).

In the case of the first material, 7SiAl, only minor differences were observed between the cooled (C) and heated (H) parts after processing, both in terms of ultimate tensile strength (UTS)—with a maximum difference of up to 60 MPa—and elongation, with a difference of up to 2%. All values are presented in Figure 10. For samples subjected to subsequent tempering (T) at 300 °C for 2 h, a negative effect on reducing both UTS and elongation (A) was observed in all cases, except for an approximately 1% increase in elongation in the cooled part after a 30 s dwell time. While the decrease in UTS is expected, the reduction in elongation is rather surprising. For 7SiAl, it can therefore be concluded that, among the evaluated processing conditions, the most favourable is a 10-s dwell time in the tool without subsequent tempering.

**Figure 11:**
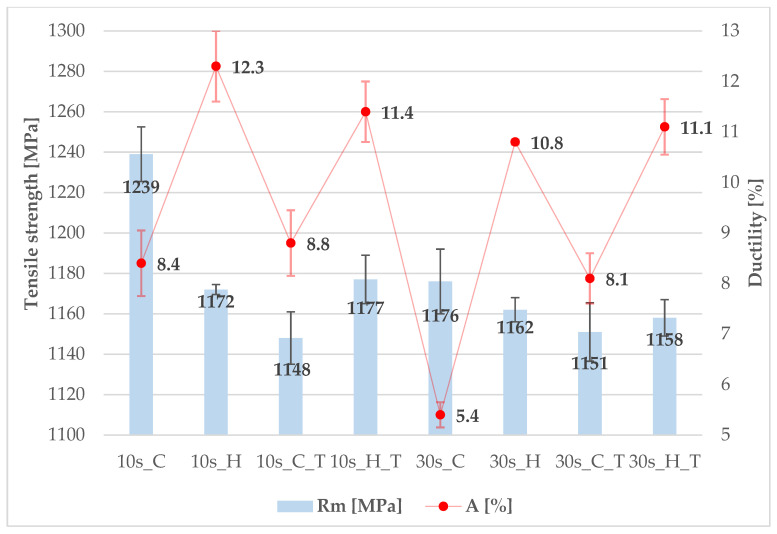
Mechanical properties of 7Al steel (cooled (C) and heated (H) part with or without tempering (T)).

Steel 7Al differs only minimally from 7SiAl in certain processing conditions with respect to ultimate tensile strength (Rm), while the differences in elongation (A) are somewhat more pronounced; even in cases with larger deviations, the UTS variation does not exceed 100 MPa. Once again, subsequent tempering does not appear to be a suitable step, as it generally leads to a decrease in UTS and, in some cases, also in elongation (A), as shown in Figure 11. An increase in elongation after tempering was observed only in the case of 30-s processing; however, this result is likely due to the unusually low elongation measured after press-hardening, which may be attributed to a local reduction in ductility caused by inclusions or other defects. In terms of mechanical properties, a dwell time of 10 s in the tool is also more favourable than a longer dwell time. Nevertheless, the comparison of 7SiAl steel and 7Al steel is more favourable for the 7Al steel, where processing with a heated tool achieves a tensile strength value above 1150 MPa and elongation above 10%. The best measured elongation value of above 12% was achieved using the heated part of the tool without subsequent tempering.

**Figure 12:**
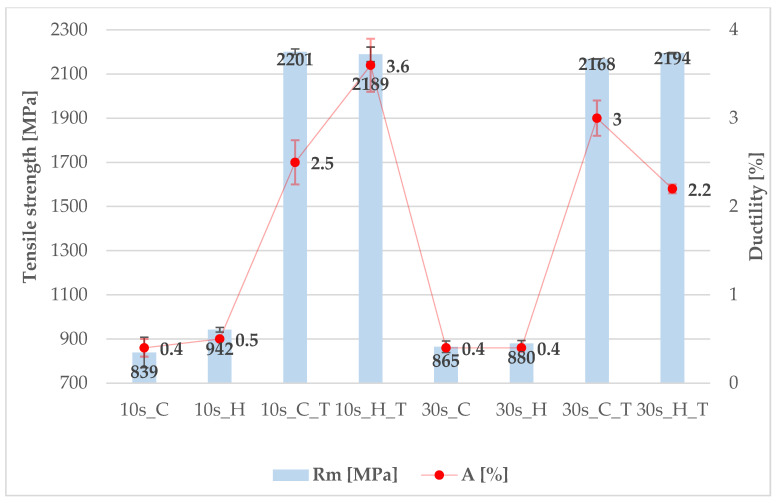
Mechanical properties of 5Al steel (cooled (C) and heated (H) part with or without tempering (T)).

The steel with a lower aluminium content—5Al—exhibits notable differences between the conditions with and without tempering. After 2 h at 300 °C, the UTS increases to approximately 2.3 times its original value (see Figure 12). In terms of elongation, the increase reaches a factor of five. In the most extreme case, the UTS rises by a factor of approximately 2.6 (from 839 MPa to 2201 MPa), while elongation increases by a factor of 7.5 (from 0.4% to 3%). Based on the measured properties, a dwell time of 10 s in the tool appears to be the most favourable option.

Table 5 shows the measured hardness HV10 for all three LDS, areas (C for cooled part; H for heated part), and heat treatment (T for tempering (300 °C, 2 h)). The HV10 hardness values are given with standard deviation. In all cases, the hardness values differ only minimally, and in some instances, the values are practically the same, since the standard deviations overlap. The differences in HV10 hardness values are minor (typically ≤5%) and remain within the standard deviations; therefore, no significant trend could be established.

When comparing the measured hardness values with the observed trends in UTS, it can be concluded that the trends are largely consistent for steels with a higher aluminium content (i.e., 7SiAl and 7Al). However, 5Al steel shows a different behaviour—its hardness measurements remain relatively constant across all conditions, which does not align with the results from the mini-tensile tests. This discrepancy suggests that, although the processed sheet likely exhibits high UTS, it fails prematurely during tensile testing due to its brittleness (i.e., low ductility/elongation). In this case, the application of tempering is highly beneficial.

## 4. Discussion

Although fully tailored properties—defined as significant mechanical property gradients between heated and cooled areas—were not entirely achieved, the experimental programme demonstrated that all listed LDSs can be processed in sheet form using press-hardening technology without the occurrence of defects. Table 6 shows the most significant mechanical values obtained after the press-hardening treatment.

### 4.1. 7SiAl and 7Al

For steels 7SiAl and 7Al, very specific microstructural features can be observed. The 7SiAl steel was not subjected to all the experimental regimes due to its similar microstructure and lower mechanical properties compared to 7Al steel. The microstructural evolution during any processing route is strongly affected by the stability of the high-temperature δ-ferrite phase. A significant fraction of this phase remains in the structure during cooling, whether in the as-cast, forged, or heat-treated condition. Its presence influences element segregation not only during solidification but also in nearly all subsequent processing steps, affects formability, and fundamentally alters the choice of austenitization temperature for any hardening treatment—including thermomechanical hot stamping. Full austenitization can only be attained with considerable difficulty at temperatures exceeding 1100 °C. At these temperatures, extensive austenite grain coarsening develops, thereby limiting the effectiveness of quenching. Retention of δ-ferrite is unavoidable; however, its presence can be used to benefit the material’s performance.

After various hot stamping regimes, microstructural analyses showed that this process in steels containing 7% Al does not lead to a quenched or tempered structure. Even cooling in the die did not produce martensitic or other hardened microstructures. Instead, under all achievable cooling rates, a ferritic–austenitic structure with a small fraction of carbides was obtained. The austenite fraction ranged between 42–55 vol.%, while the remainder of the material consisted predominantly of δ-ferrite. These phase fractions were confirmed both by metallographic observations and by X-ray diffraction quantification. Carbides represented only a minor portion of the structure, either formed by the eutectoid decomposition of austenite or by direct precipitation of discrete carbide particles. In the case of the eutectoid transformation, additional ferrite was introduced into the structure. In high-Al steels, the heated part of the tool showed a higher proportion of eutectoid decomposition products (pearlite) due to extended residence in the pearlitic region during cooling. Notably, although the structure cannot be classified as hardened, it still exhibited high strength—consistently above 1100 MPa. This is most likely promoted by relatively fine austenite grains and the large interfacial area between ferrite and austenite. The elongation of approx. 10–11% is a promising result, although higher values would be necessary for many anticipated applications. In the heated part of the tool, ductility increases significantly, accompanied by a slight increase in the amount of carbides.

The observed stability of austenite in these steels can be explained by assuming carbon partitioning into the austenite regions, promoted by the large amount of δ-ferrite. When cooled at specific critical rates, carbon-enriched austenite remains stable down to room temperature. Martensitic transformation would occur at higher cooling rates, as reported in [6], whereas decomposition into ferritic–carbide structures would occur at lower cooling rates [3]. However, austenite remains retained and stable in ambient conditions within the critical cooling range. Furthermore, the experimental programme demonstrated that austenite in these steels remains stable even after tempering up to 300 °C, with complete decomposition—as suggested by tempering dilatometry (Figure 3)—occurring only at temperatures around 500 °C or higher.

Interesting phenomena can be observed during optimising the hot stamping tool temperature, dwell time in the tool, and introducing tempering after hot stamping. Processing in the heated tool provides consistently better results compared to the cooled tool, probably due to lower internal stress, which remains in the material after hot stamping. The different dwell times in the tool of 10 and 30 s had almost no effect on the microstructures, but a longer dwell time led to a decrease in the tensile strength and ductility in both the cooled and heated parts of the omega profile. Due to the achievable cooling rates in the cooled part of the tool, martensitic structures could not be obtained, and higher quench rates would be required for hardened microstructures. The introduction of low temperature tempering seems to have a harmful effect on both strength and plasticity parameters. Due to the absence of martensite in the microstructure, tempering primarily affects the onset of austenite decomposition. For both steels, the expected beneficial effect of tempering has not yet been achieved. This is influenced by the presence of a duplex structure without martensite. Further optimisation of the tempering process may yield more favourable results. The formation of new phases during tempering—primarily through austenite decomposition—and their impact on the system’s properties remain unclear. This represents another potential direction for the research programme. Specific detrimental effects may occur that are not yet visible in the microstructure.

These characteristics mean that steels containing 7% Al can be characterised as duplex steels, with approximately 50% stable austenite. Another way to achieve a high RA percentage is to use intercritical annealing; e.g., in [28], over 35% RA is achieved, but a steel with a different chemical composition is used. Similar duplex-type structures are also reported for other alloy systems, such as super duplex stainless steel UNS S32750 (≈25% Cr, 3–5% Mo, 6–8% Ni, 0.24–0.32% N) [29] and duplex stainless steel 2205 (≈22% Cr, 3% Mo, 5–6% Ni) [30]. Although similar duplex-type structures (austenite + δ-ferrite) are reported in the literature, comparable microstructures have not yet been described for high-Al steels of the type investigated in this study. 7SiAl and 7Al exhibited a rare ferrite–austenite duplex microstructure for steels of this chemical composition, with high austenite stability and a low fraction of carbides. Due to the extensive austenite–ferrite interfacial area and the synergistic interaction between the coexisting phases, duplex steels are known to possess unique properties that are exploitable in various applications. This type of microstructure is suitable for a wide range of applications due to its expected combination of favourable mechanical properties, corrosion resistance, thermal stability, high energy absorption capacity, and reduced density. Some of these assumptions have already been confirmed through the experimental programme, such as favourable UTS (~1100 MPa) and elongation (~10%), and reduced density of the sheets (~7.08–7.18 g/cm^3^, ~9% lower than conventional 22MnB5). Other properties, such as ductility, require further optimisation. Further improvements may also be achieved by refining the microstructure, particularly by subdividing the elongated ferritic regions into smaller grains. Corrosion resistance, thermal stability, and energy absorption capacity have not yet been measured and must be verified. Typical examples include components exposed to corrosive environments, parts requiring high damping capacity, or components where a favourable balance between strength and ductility is essential. The 9% reduction in density represents an additional advantage that may further support its industrial implementation.

The current property set opens opportunities for applications where high ductility is not the primary requirement. Considering the benefit of reduced density, logical application areas include the automotive industry, where chassis components, for instance, could take advantage of the combination of properties. Nevertheless, the dataset of properties must be complemented by toughness, fatigue testing, and possibly other parameters. For any potential use in lightweight, high-strength body-in-white components, a substantial improvement in ductility would be necessary, together with additional testing such as hole expansion and bulge tests. Nevertheless, even with the current properties, these steels can be used for lightweight, less mechanically demanding components. However, the present results already demonstrate that these steels are suitable for further development and for selected applications in sheet and in other semi-finished forms.

### 4.2. 5Al

5Al steel can also be processed by press-hardening without macroscopic defects in critical regions. However, in terms of structural integrity, metallographic analysis revealed problematic sites, particularly along prior austenite grain (PAG) boundaries. These boundaries are highly etchable, suggesting potentially weak regions in the microstructure. In some locations, the etched area extended to such an extent that it could be interpreted as the initiation of a crack. The weakening of these boundaries may have different origins; the most likely explanation is the segregation of certain alloying elements, whose excessive presence at the boundary locally reduced cohesion. Alternatively, undesired reactions may have taken place at these interfaces, resulting in the formation of intermediate phases.

The matrix of hot-stamped 5Al steel consisted predominantly of lath martensite in all processing conditions, but contained a relatively high fraction of retained austenite while δ-ferrite was nearly absent. X-ray diffraction analysis revealed that approximately 30% of RA was present after press hardening. Following subsequent tempering, this amount decreased by about half, reaching 15%. The martensite laths were organised into packets, and clusters of these packets occupied the volume of the prior austenite grains. Under loading, the weakest regions of this structure are likely the PAG boundaries. Improving the quality of this steel and, in particular, enhancing its ductility, would therefore require the elimination of these unfavourable boundary phenomena. Further progress will depend on the optimisation of the chemical composition and processing conditions.

The currently measured mechanical properties demonstrate an exceptionally large difference between the ultimate tensile strength after die quenching and after tempering. An increase of more than 1000 MPa is highly unusual for quenched-and-tempered steels. It is most likely associated with the decomposition of a large fraction of the retained austenite and its transformation into a martensitic–carbide structure. In addition, precipitation hardening from transit carbides can be assumed. A similar correlation has already been demonstrated in [6] and can also be observed in Figure 3. It is also noteworthy that, in addition to the remarkable increase in strength, ductility improved several-fold after tempering. However, this increase reflects a rise from extremely low values (<1%) to a maximum of 3.6%. The simultaneous increase in strength and ductility after tempering suggests at least a partial recovery of the defective microstructure in the as-quenched condition.

If the formation of defects at PAG boundaries can be eliminated, the use of 5Al steel would be attractive for components requiring high hardness and wear resistance, where high ductility is not essential. In terms of shape, the material appears more suitable for bulk components or parts manufactured from thicker sheets. In any case, further development should be directed toward improving the ductility of this steel. One promising approach is the refinement of the relatively coarse martensitic structure, which may contribute to enhanced toughness. Moreover, particular attention should be paid to the nature and quality of the decomposition products of retained austenite, as these significantly influence the final mechanical properties.

Microstructural evolution was largely independent of dwell time: both 10 s and 30 s cycles resulted in comparable phase fractions and hardness levels. For practical purposes, a dwell time of 10 s is sufficient, reducing process time without loss of performance.

In addition to its mechanical potential, 5Al steel offers very high achievable strength and hardness, making it suitable for applications where wear resistance and reduced weight are critical. Although its current applicability is limited by insufficient ductility and toughness, further optimisation may render it a competitive material. Similar to 7Al, promising corrosion resistance and thermal stability are expected, but these assumptions require experimental validation.

## 5. Conclusions

The key findings of the experiments are summarised as follows:

- All investigated high-carbon low-density steels (7SiAl, 7Al, 5Al) can be processed by press hardening without macroscopic defects.
- All LDS variants exhibited a density reduction of ~9% compared to conventional press-hardening steel 22MnB5 (7.08–7.18 g/cm^3^ vs. ~7.8 g/cm^3^). This weight saving represents a significant advantage for automotive body structures and components aimed at reducing fuel consumption and CO_2_ emissions.
- Steels with 7% Al (7Al and 7SiAl) exhibit a rare duplex ferrite–austenite microstructure for steels of this chemical composition, achieving UTS ≈ 1100–1200 MPa and elongation up to 12%.
- Steel 5Al forms a martensitic matrix with retained austenite; after tempering at 300 °C for 2 h, UTS exceeds 2200 MPa, but ductility remains low (≤3.6%).
- Dwell time (10 vs. 30 s) has a negligible effect; 10 s is sufficient for practical processing.
- Tempering improves properties only for 5Al; for 7Al and 7SiAl, it does not provide benefits.
- This study provides the first systematic evaluation of press-hardening for Fe–Al–C steels with high carbon and reduced manganese, addressing a gap left by previous research and delivering comparative data for three compositions (7SiAl, 7Al, 5Al).

## Figures and Tables

**Table 1:** Chemical composition of LDS (wt. %) [6].

Material	C	Si	Mn	Cr	Ni	Al	S	P
7SiAl (7%Al)	0.69	0.58	0.53	2.06	1.004	7.06	0.007	0.01
7Al (7%Al)	0.73	0.12	0.55	2.02	1.05	7.06	0.008	0.01
5Al (5%Al)	1.07	0.16	0.53	2.03	1.56	4.97	0.006	0.008

**Table 2:** Parameters of processing in the small omega tool.

Materials	Furnace Temp. [°C]	Soaking Time [min]	Tool Temp. [°C]	Time in the Tool [s]	Heat Treatment
7SiAl; 7Al; 5Al	950	10	20	30	300 °C; 2 h
			20	30	600 °C, 2 h
			300	1800	cooled in water
			20	30	600 °C, 1 h

**Table 3:** Parameters of processing in the small tool with the selected regime.

Material	Rm [MPa]	Rp0.2 [MPa]	A [%]	HV10
7SiAl	1109 ± 17	830 ± 7	7.5	358 ± 6
7Al	1122 ± 7	729 ± 4	8.3	332 ± 2
5Al	2023 ± 4	1545 ± 2	6.6	589 ± 14

**Table 4:** Parameters of processing in the combined tool.

Material	Furnace Temp. [°C]	Soaking Time [min]	Tool Parts [°C]		Time in the Tool [s]
			Heated	Cooled	
7SiAl	950	10	250	20	10; 30
7Al	950	10	285	20	10; 30
5Al	950	10	50	20	10; 30

**Table 5:** Hardness of all three experimental low-density steels.

Material	Sample	HV10	Sample	HV10	Sample	HV10	Sample	HV10
7SiAl	10s_C	355 ± 2	10s_C_T	353 ± 2	30s_C	354 ± 2	30s_C_T	349 ± 1
	10s_H	375 ± 2	10s_H_T	372 ± 1	30s_H	363 ± 7	30s_H_T	360 ± 3
7Al	10s_C	345 ± 3	10s_C_T	332 ± 1	30s_C	342 ± 6	30s_C_T	334 ± 4
	10s_H	348 ± 2	10s_H_T	342 ± 5	30s_H	347 ± 2	30s_H_T	340 ± 4
5Al	10s_C	660 ± 6	10s_C_T	692 ± 1	30s_C	670 ± 3	30s_C_T	659 ± 2
	10s_H	666 ± 3	10s_H_T	700 ± 6	30s_H	658 ± 3	30s_H_T	658 ± 4

**Table 6:** Summary of the most significant results obtained after press-hardening for each material.

Material	Part	Rm [MPa]	A [%]	HV10	Regime
7SiAl	C	1144	8.4	355	10 s
	H	1201	10.2	375	
7Al	C	1239	8.4	345	10 s
	H	1172	12.3	348	
5Al	C	2201	2.5	692	10 s + T
	H	2189	3.6	700	

## Data Availability

The original contributions presented in this study are included in the article. Further inquiries can be directed to the corresponding author.
